# Transcriptome Analysis of *Pyrus betulaefolia* Seedling Root Responses to Short-Term Potassium Deficiency

**DOI:** 10.3390/ijms21228857

**Published:** 2020-11-23

**Authors:** Han Yang, Yan Li, Yumeng Jin, Liping Kan, Changwei Shen, Anish Malladi, Savithri Nambeesan, Yangchun Xu, Caixia Dong

**Affiliations:** 1 Jiangsu Provincial Key Lab of Solid Organic Waste Utilization, Jiangsu Collaborative Innovation Center of Solid Organic Wastes, Educational Ministry Engineering Center of Resource-Saving Fertilizers, Nanjing Agricultural University, Nanjing 210095, China; 2017103095@njau.edu.cn (H.Y.); 2018103099@njau.edu.cn (Y.J.); 2019103095@njau.edu.cn (L.K.); ycxu@njau.edu.cn (Y.X.); 2 College of Life Science, Hubei Engineering University, Xiaogan 432100, China; liyan20089002@163.com; 3 School of Resources and Environmental Sciences, Henan Institute of Science and Technology, Xinxiang 453003, China; changweishen@163.com; 4 Department of Horticulture, 1111 Miller Plant Sciences, University of Georgia, Athens, GA 30602, USA; malladi@uga.edu (A.M.); sunamb@uga.edu (S.N.)

**Keywords:** pear, potassium deficiency, root, potassium transporters and channels, transcriptome analysis, RNA-seq, DEGs

## Abstract

Potassium (K) plays a crucial role in multiple physiological and developmental processes in plants. Its deficiency is a common abiotic stress that inhibits plant growth and reduces crop productivity. A better understanding of the mechanisms involved in plant responses to low K could help to improve the efficiency of K use in plants. However, such responses remain poorly characterized in fruit tree species such as pears (*Pyrus* sp). We analyzed the physiological and transcriptome responses of a commonly used pear rootstock, *Pyrus betulaefolia*, to K-deficiency stress (0 mM). Potassium deprivation resulted in apparent changes in root morphology, with short-term low-K stress resulting in rapidly enhanced root growth. Transcriptome analyses indicated that the root transcriptome was coordinately altered within 6 h after K deprivation, a process that continued until 15 d after treatment. Potassium deprivation resulted in the enhanced expression (up to 5-fold) of a putative high-affinity K^+^ transporter, *PbHAK5* (*Pbr037826.1*), suggesting the up-regulation of mechanisms associated with K^+^ acquisition. The enhanced root growth in response to K-deficiency stress was associated with a rapid and sustained decrease in the expression of a transcription factor, *PbMYB44 (Pbr015309.1)*, potentially involved in mediating auxin responses, and the increased expression of multiple genes associated with regulating root growth. The concentrations of several phytohormones including indoleacetic acid (IAA), ABA, ETH, gibberellin (GA_3_), and jasmonic acid (JA) were higher in response to K deprivation. Furthermore, genes coding for enzymes associated with carbon metabolism such as *SORBITOL DEHYDROGENASE (SDH)* and *SUCROSE SYNTHASE (SUS)* displayed greatly enhanced expression in the roots under K deprivation, presumably indicating enhanced metabolism to meet the increased energy demands for growth and K^+^ acquisition. Together, these data suggest that K deprivation in *P. betulaefolia* results in the rapid re-programming of the transcriptome to enhance root growth and K^+^ acquisition. These data provide key insights into the molecular basis for understanding low-K-tolerance mechanisms in pears and in other related fruit trees and identifying potential candidates that warrant further analyses.

## 1. Introduction

Potassium (K) is the most abundant monovalent cation in plant cells [1]. It plays a crucial role in many physiological processes in plants, including osmoregulation, the control of turgor pressure, electrical neutralization, and enzyme activation [2]. The K^+^ concentration in a soil solution generally varies from 0.02 to 5 mM, while the K^+^ concentration in the rhizosphere is usually less than 0.3 mM [3,4]. Therefore, many plants are subject to low-K stress at some phase of their life cycle. Potassium deficiency in plants often manifests as reduced photosynthesis and reduced primary-root growth [5]. In plants displaying low-K tolerance, a short period of K-deficiency stress leads to the promotion of plant growth, referred to as the stress-induced morphogenic response (SIMR) [6]. Sustained root growth to enhance the root surface area, increase the efficiency of K^+^ acquisition, and enhance internal translocation (including re-mobilization) enables plants to respond to low-K stress [6]. 

The responses to K deficiency vary across species and across genotypes within a species [6]. Zhao [7] screened different watermelon genotypes with low-K tolerance and found that short-term K-deficiency treatment had no negative effects on the plants. Sun [8] demonstrated that low-K treatment inhibited the growth of an apple rootstock (*Malus xiaojinensis*), allowing it to tolerate low K. Furthermore, low K had little effect on the length of banana primary roots but reduced root hairs [9]. Low-K stress reduced the number of lateral roots of tomatoes and increased root hairs in a K-efficient genotype of tomatoes [10]. After the low-K stress treatment of maize seedlings, Ning et al. [11] found that the root length of plants tolerant to K deficiency increased significantly. Two tobacco genotypes displayed differing root and shoot growth, and K-homeostasis responses during low-K stress [12]. The genotype tolerant to low K displayed a reduced inhibition of first-order lateral-root growth under K-deficiency stress. 

Under low-K stress, plants display multiple root-related phenotypes including the inhibition of primary-root growth, the promotion of root-hair extension, and an increase in the root surface area by regulating the morphology and structure of plant roots [13,14]. Root morphogenetic responses under K deficiency are partly mediated by phytohormone regulation [13,15,16]. Long-term K deficiency can lead to a decrease in IAA (indoleacetic acid) and GA_3_ (gibberellin) concentrations and the accelerated senescence of plants [7]. Low-K treatment results in increased ethylene production, which subsequently alters reactive oxygen species (ROS) production and regulates root growth and K^+^ acquisition [17,18]. The exogenous application of ethylene and auxin inhibits the growth of primary roots, similar to responses under low-K stress, suggesting an interaction between these hormones and K deficiency [17]. Furthermore, K deficiency enhances the accumulation of ABA in roots and its long-distance transport, which induces the production of ROS and stomatal closure [19]. Additionally, the accumulation of JA (jasmonic acid) under low-K stress can enhance plant defense responses [20]. 

Potassium acquisition in plants is mediated by high-affinity and low-affinity transport systems. A high-affinity K^+^-uptake system in roots, especially involving the high-affinity K^+^ transporters and K^+^ channels [21,22], allows most plants to acquire K^+^ under low-K conditions. Different plant species or different genotypes within a species have diverse K^+^-uptake efficiencies [23]. Previous studies reported that high-affinity K^+^ transporter genes were induced by low-K conditions. For example, several members of the HAK/KUP/KT (high-affinity K^+^/K^+^ uptake/K^+^ transporters) family including *AtHAK5* [24], *HvHAK1* [25], and *OsHAK1* [26] as well as the K^+^ channel genes *AtAKT1* [27] and *AtCHX17* [28] were induced by K deficiency. The induction of these transport systems constitutes an important mechanism by which plants continue K^+^ acquisition and acclimate to K-deprivation stress. 

Transcription factors from several families such as MYB and AP2 play important roles in root development and abiotic stress responses including K deficiency [29]. In *Arabidopsis thaliana*, MYB77 interacts with AUXIN RESPONSE FACTORS (ARFs) to enhance the auxin response [30]. In Arabidopsis, MYB77 interacts with ARF7 to facilitate a significant reduction in the number of lateral roots. At the same time, *ARF2* acts as an inhibitor of *HAK5* by regulating its transcription [31]. The ethylene-induced ROS pathway enhances K^+^ absorption, potentially through the interaction of AP2/ERFs with *HAK5* [32], but its mechanism is still unclear. The *RAP2.3 (RELATED TO AP2.3)* transcription factor belongs to the AP2/ERF family and is induced by ethylene [33]. It can increase tolerance to oxidative and osmotic stress through ABA-mediated osmotic regulation [34]. In Arabidopsis, *RAP2.11* expression is induced under K deprivation, and the protein interacts with the *HAK5* promoter to positively regulate its expression, along with that of other low-K responsive genes [35]. 

A substantial proportion of agricultural soils worldwide display deficient K levels owing to various factors, including continuous crop production [36]. The sustainable and profitable production of crops, including fruit trees such as pears (*Pyrus* sp.), is dependent upon increasing their nutrient-use efficiencies, such as those of K. To achieve this goal, a better understanding of K^+^ uptake and utilization is essential. In many cultivated fruit trees such as pears, nutrient utilization and deficiency responses are additionally dependent on the rootstock utilized. *Pyrus betulaefolia* is a common pear rootstock that is widely used across the world and displays several disease resistance and nutritional efficiency characteristics. Transcriptomic analyses of *P. betulaefolia* responses to K deficiency can contribute greatly to our understanding of the molecular mechanisms underlying the responses to K limitation in these plants. In the present study, we analyzed the physiological, morphogenic, and transcriptomic responses of *P. betulaefolia* seedling roots during K deprivation. We used the approach of complete K deprivation, as it allows for a better understanding of the immediate and short-term responses to K-deficiency stress in a perennial fruit tree system. 

## 2. Results

### 2.1. Short-Term K Deficiency Enhances Root Growth of P. betulaefolia Seedlings

**Figure 1:**
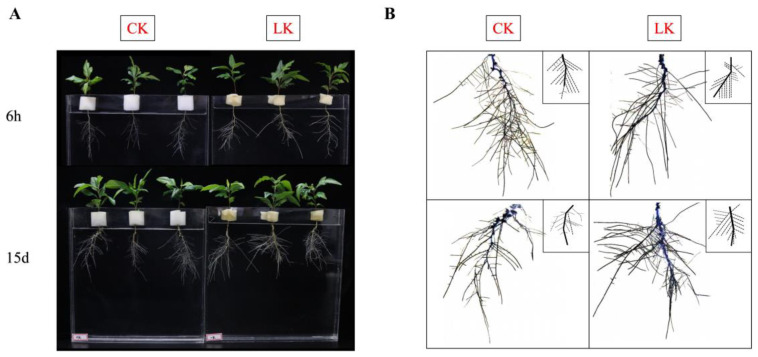
Effects of K availability on root morphology of *P. betulaefolia* seedlings. (**A**) CK (control) and LK (no K^+^) treatment at 6 h and 15 d after K^+^ deprivation. (**B**) Root system architecture at 15 d after K^+^ deprivation in *P. betulaefolia* seedlings.

Low-K stress significantly increased the root length (63%), root surface area (108%), root diameter (163%), root volume (191%), and number of root tips (130%) (Table 1; Figure 1A). Simultaneously, it increased the angle between the lateral root and the main root (Figure 1B). 

### 2.2. K Deprivation Affects Remobilization, Rapidly Decreases Medium pH, and Affects N Acquisition

**Figure 2:**
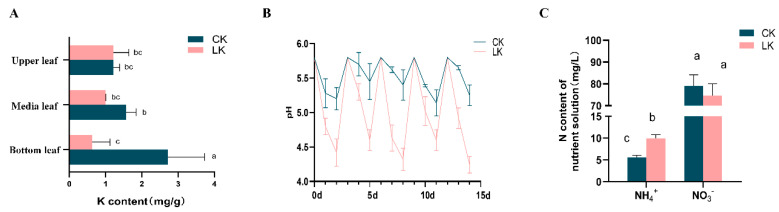
Effects of K deprivation in *P. betulaefolia* seedlings on nutrient concentration in the plant and on medium characteristics. (**A**) K^+^ concentration in leaves from different parts of the *P. betulaefolia* plants at 15 d after K deprivation. (**B**) pH change in the medium after imposition of K^+^ deprivation. (**C**) The concentration of NH_4_^+^ and NO_3_^-^ in the medium at 15 d after K deprivation. Error bars represent standard error (*n* = 6). CK: Control; LK: Low-K treatment. Similar letters associated with the bars indicate that the values are not significantly different.

Potassium is highly mobile within the plant and can quickly move to new and actively growing regions. Consistently, the K^+^ concentration in old leaves was reduced by over 5-fold under K deprivation, but no difference in K^+^ concentration was observed in newer leaves (Figure 2A). The pH in the growth media was maintained close to 5.8 by the addition of NaOH (1M) every three days. Following correction, the medium pH consistently dropped within 1–2 d by around 0.5 units in the control (Figure 2B). The drop in pH was greater, by about 1 unit, under K deficiency than that in the control (Figure 2B). It is worth noting that, even if there were no visual symptoms under K deficiency for 15 d, elemental analyses indicated that the plants were deficient in K, and root morphological analysis indicated the promotion of root growth. To determine if the rapid absorption of nitrogen (N) was associated with the promotion of root growth, the concentrations of NH_4_^+^ and NO_3_^−^ in the medium at 15 d after K deprivation were measured (Figure 2C). The NH_4_^+^ concentration of the medium was significantly higher with the low-K treatment than that in the control, while the concentration of NO_3_^−^ was not different across the treatments. The overall total N concentration of the plant did not change significantly. 

### 2.3. Transcriptome Sequencing Identified Differentially Expressed Genes (DEGs) in P. betulaefolia Seedlings under Low-K Stress

An Illumina HiSeq 2500 (San Diego, CA, USA) was used to conduct the high-throughput transcriptome analysis of control and low-K-stress-treated root samples at 6 h and 15 d after treatment. Around 41,070,000 and 57,870,000 clean reads were obtained, respectively, and accounted for over 98% of the total sequences (Table S1). FASTQC was used to assess the sequence quality in both the control and treatment samples. The transcriptome analysis was conducted using the complete genomic sequence of Chinese white pears (*P. bretschneideri*) as a reference. Most of the gene sequences were well matched with the reference genome (Table S1). The majority of the matched genes had a coverage of 67.95–75.36%. 

**Figure 3:**
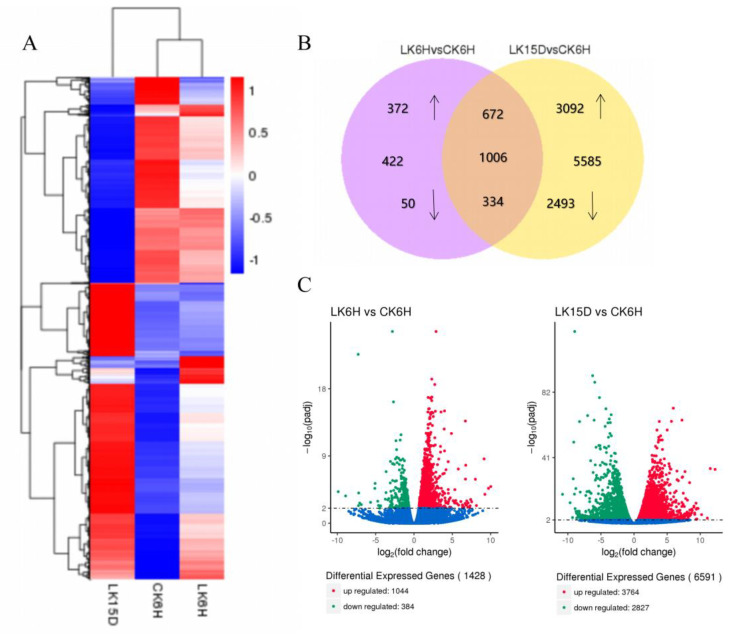
Transcriptome analysis of *P. betulaefolia* roots under K stress. A–C: (**A**) Hierarchical clustering of all differentially expressed genes (DEGs) showing distinct gene expression profiles in the pear root. Different columns represent the different samples. (**B**) Venn diagram of expressed genes (FPKM ≥ 2; Fragments per kilobase per million reads) for each library (6 h: 1428 genes; 15 d: 6591 genes). The number of commonly expressed genes in each intersection area is presented. (**C**) Volcano plots of DEGs. The *x*-axis shows the fold change in gene expression, and the y-axis shows the statistical significance of the differences. Blue dots indicate genes without significant differential expression. The red dots show significantly up-regulated DEGs, and the green dots, down-regulated DEGs. CK: Control; LK: Low-K treatment.

The reads per kilobase of transcript per million mapped reads (RPKM) calculation method was employed to compare transcriptome differences between the control and the treated group. This study yielded a total of 1428 differentially expressed genes at 6 h after K deprivation, of which 1044 genes were up-regulated and 384 genes were down-regulated (Figure 3; Table S4). Around 6591 genes were differentially expressed at 15 d after K deprivation, of which 3764 were up-regulated and 2827 were down-regulated (Figure 3; Table S5). Compared with 6 h after low-K stress, there were 672 up-regulated genes and 334 down-regulated genes in the roots at 15 d after low-K stress (Figure 3). The results indicate a strong correlation between the RNA-seq and qPCR data (R^2^ = 0.93; Figure S1). We identified 73 candidate genes involved in the low-K response under K deprivation (Figure 3; Table S2) from 1006 genes displaying common differences across the two periods.

### 2.4. Functional Classification of DEGs Identified in Low-K Conditions

**Figure 4:**
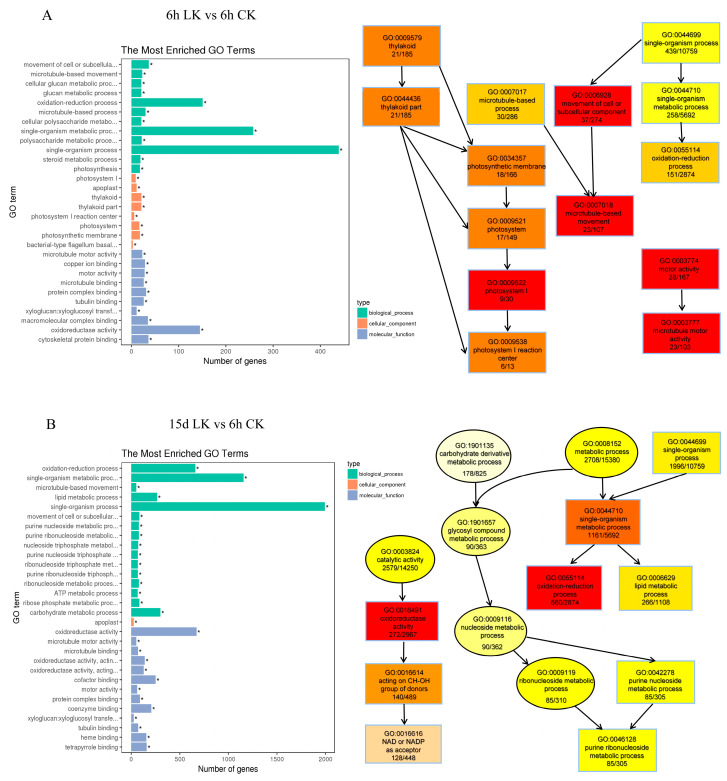
GO enrichment analysis of co-regulated DEGs detected in roots in K deficiency for 6 h (**A**) and 15 d (**B**). Functional categorization of genes based on the biological process of gene ontology. CK: Control; LK: Low-K treatment. Asterisk indicates an enriched GO category.

**Figure 5:**
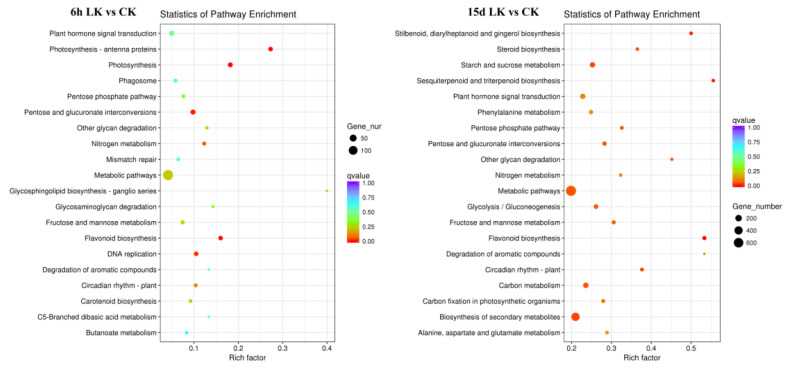
Kyoto Encyclopedia of Genes and Genomes (KEGG) enrichment analysis of DEGs in roots. A larger Rich factor indicates a higher degree of DEG enrichment. The value range of the Q value is from 0 to 1, which means that the significance of the DEG enrichment declines from 0 to 1. CK: Control; LK: Low-K treatment.

To better understand low-K-related responses, we used GoPipe to conduct GO classification on the differentially expressed genes. A total of 1820 predicted proteins were matched with 2298 GO terms at 6 h after K deprivation, while a total of 1843 predicted proteins were matched with 2278 GO terms at 15 d after K deprivation. GO classification includes molecular function (MF), biological process (BP), and cell composition (CC) processes (Figure 4; Tables S8 and S9). GO enrichment showed that genes with functions related to catalytic activity, binding activity, and nutrient storage were up- and down-regulated under short-term low-K stress (6 h). The biological processes that these genes primarily participated in involved metabolism, growth and development, multicellular processes, and signal transduction processes. Among the molecular functions, genes that encoded proteins with binding and catalytic activity accounted for a large portion under low-K stress. In addition, many genes participated in cell growth and metabolism, and their expression was stimulated by long-term low-K stress. Kyoto Encyclopedia of Genes and Genomes (KEGG) analysis also showed that the DEGs were primarily involved in metabolic pathways and the biosynthesis of secondary metabolites (Figure 5, Tables S6 and S7). 

### 2.5. DEGs Related to Phytohormones

**Figure 6:**
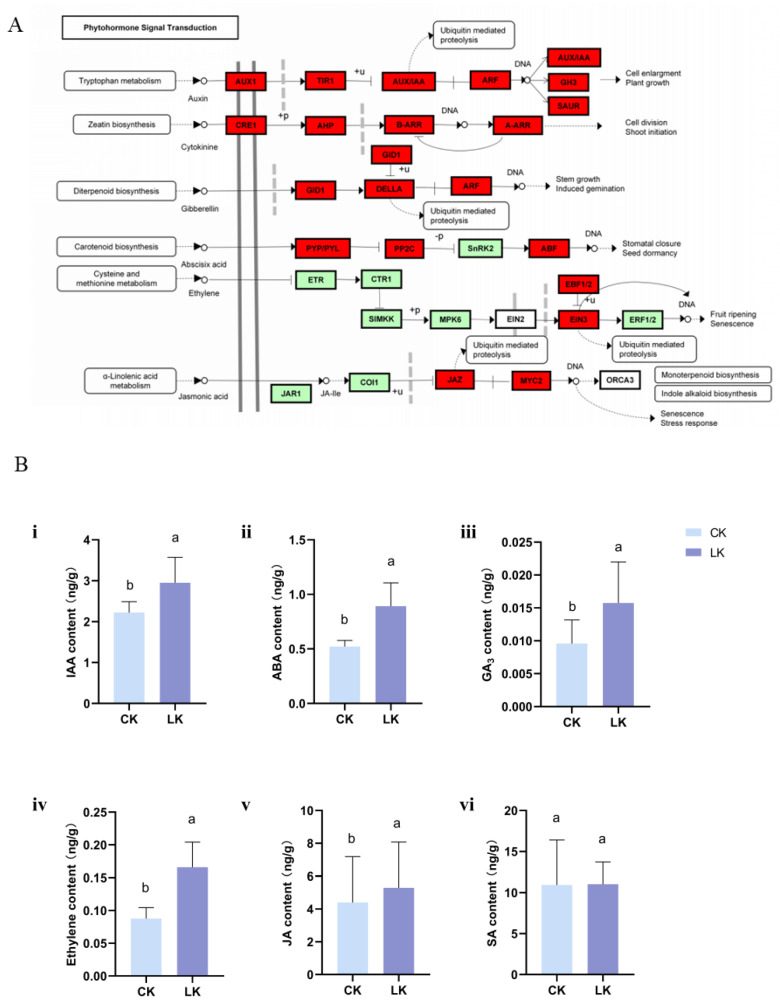
KEGG enrichment analysis of phytohormone metabolism and signaling in roots (**A**), and the analysis of phytohormone concentrations in response to low-K stress (**B**). A: Red and green boxes indicate phytohormone metabolism and signaling pathway-related genes that were up- or down-regulated based on the transcriptome analysis. (**B**): (**i**) indoleacetic acid (IAA) concentration; (**ii**) ABA concentration; (**iii**) gibberellin (GA_3_) concentration; (**iv**) ETH concentration; (**v**) jasmonic acid (JA) concentration; (**vi**) SA concentration. Error bars represent standard error (*n* = 6). CK: Control; LK: Low-K treatment. Similar letters after numbers indicate that values are not significantly different within the column.

Multiple genes associated with phytohormone biosynthesis and signaling, such as that of ethylene, auxin, gibberellin, and JA, were differentially expressed in response to low-K stress (Figure 4 and Figure 6A). Analyses of phytohormone concentrations at 15 d after K deprivation indicated significantly increased concentrations of ABA and GA_3_ by up to around 1.5-fold in the roots. Furthermore, the concentration of endogenous ETH in the root increased by >2-fold under K deficiency (Figure 6B).

### 2.6. DEGs Encoding Transporters

**Figure 7:**
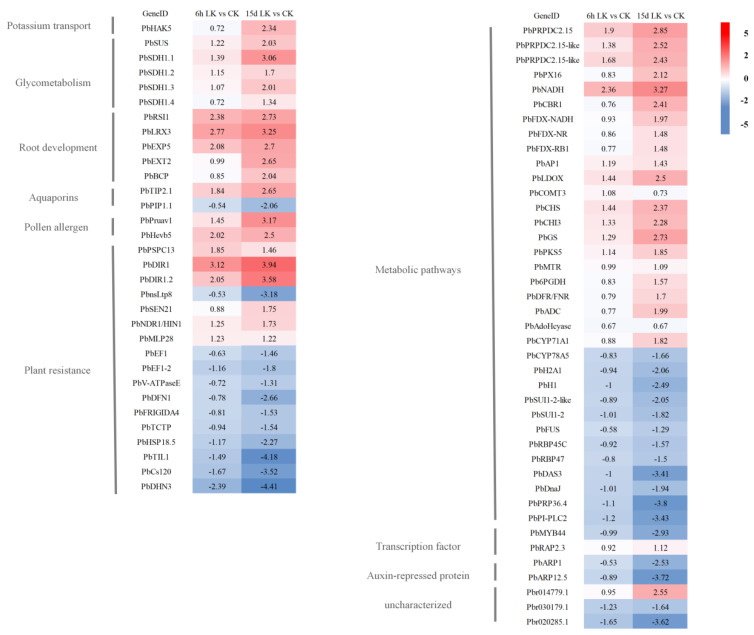
Differential gene expression in *P. betulaefolia* roots under low-K stress. Differentially expressed genes that were commonly regulated at 6 h and 15 d after K^+^ deprivation are presented. The color scale represents FPKM-normalized log2-transformed gene expression levels. The value of log2 fold change is presented for each gene at each stage after K deprivation. LK: Low-K treatment; CK: Control.

**Figure 8:**
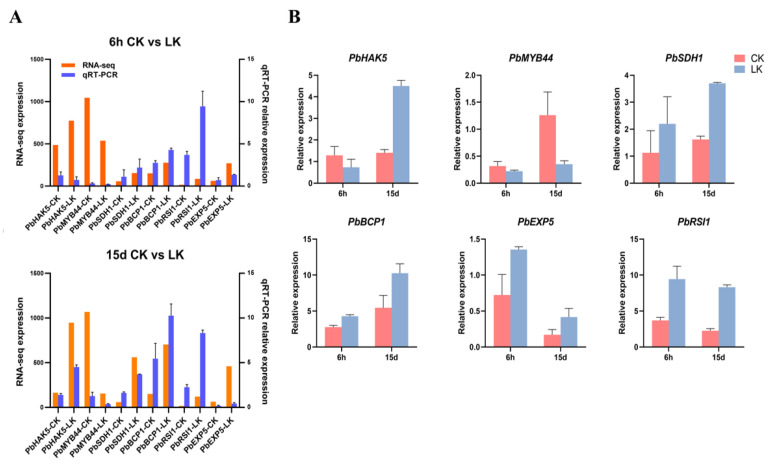
Relative gene expression analysis using quantitative RT-PCR. (**A**): Comparison of gene expression values obtained from RNA-seq data and those obtained using quantitative RT-PCR at 6 h and 15 d after K deprivation. (**B**): Quantitative RT-PCR data of a sub-set of genes differentially expressed in the root in response to low-K stress. CK: Control; LK: Low-K treatment. The experiments were repeated three times. The error bars represent mean ± SE (*n* = 3).

The absorption and translocation of K^+^ in plants occur mainly via high- and low-affinity K^+^-uptake systems, which are mediated by K^+^ transporters and channels. A putative high-affinity transporter, *PbHAK5 (Pbr037826.1),* was up-regulated by more than 5-fold at 15 d after treatment in response to low-K stress (Figure 7; Tables S2 and S5). Real-time PCR analyses further confirmed that the expression of *PbHAK5* was significantly up-regulated under K-deficiency conditions by >3-fold at 15 d after treatment (Figure 8). In addition to K^+^ transporter genes, we found that the level of the transcripts of genes involved in N transport was altered in pear roots during K deficiency. The nitrate-transport-related *NRT2* genes (*Pbr030334.1* and *Pbr030335.1*) were up-regulated after 6 h (up to 3.5-fold) and 15 d (up to 7.3-fold) of exposure to low-K stress (Tables S2, S4 and S5). 

### 2.7. DEGs Encoding Transcription Factors

Around 119 genes encoding transcription factors were identified within the DEGs (Tables S4 and S5). The transcription factor families regulated by low-K stress in the pear roots mainly included MYB, AP2/EREBP, bHLH, zinc finger, NAC, bZIP, AUX/IAA, and ARF, suggesting that they might play vital roles in the low-K stress response. Through the analysis of common differences between the two time periods, *PbMYB44 (Pbr015309.1)* was identified as a transcription factor displaying a reduction in expression by around 2-fold at 6 h and by over 7-fold at 15 d after K deprivation (Figure 7 and Figure 8 and Table S2). Additionally, *PbRAP2.3*, putatively encoding an ethylene-regulated transcription factor, was up-regulated by around 2-fold at 15 d after K deprivation (Figure 7; Table S2).

### 2.8. DEGs Associated with Carbohydrate Metabolism 

Analyses of genes involved in carbohydrate metabolism indicated that multiple *SDHs (SORBITOL DEHYDROGENASEs; Pbr032776.1*, *Pbr032775.1*, *Pbr013913.1*, and *Pbr032777.1)* were up-regulated by up to 2.5-fold at 6 h and by up to 8-fold at 15 d under K-deprivation conditions (Figure 7 and Figure 8 and Table S2). SDH is involved in the conversion of sorbitol, the main translocated carbohydrate in many Rosaceae plants, to fructose. Additionally, *SUS1 (SUCROSE SYNTHASE; Pbr037395.1)*, typically involved in the breakdown of sucrose to fructose and UDP-glucose, was up-regulated by 4-fold in response to K^+^ deprivation at 15 d after treatment.

### 2.9. DEGs Encoding Root Growth and Development-Associated Proteins

As root growth was enhanced in response to K deprivation, genes associated with root growth and development were further evaluated. At least five genes associated with root growth and development were found to be up-regulated in response to K deprivation (Figure 7 and Figure 8 and Table S2). Interestingly, many of these were up-regulated within 6 h after treatment and continued to increase until 15 d after treatment (Figure 7; Table S2). *PbEXP5* (*EXPANSIN 5; Pbr039073.1*), a gene putatively coding for an EXPANSIN, was up-regulated by 4- and 6-fold at 6 h and 15 d after K deprivation, respectively. Two transcripts putatively coding for EXTENSINS, *PbEXT2* (*EXTENSIN 2; Pbr031110.1*) and *PbLRX3* (*LEUCINE-RICH REPEAT EXTENSIN PROTEIN 3; Pbr016594.1*), were up-regulated by up to 6.6-fold at 6 h and by up to 9.4-fold at 15 d in the roots under K deprivation. *PbRSI1* (*ROOT SYSTEM INDUCIBLE 1; Pbr024532.1*), a gene associated with lateral-root initiation, was identified as up-regulated in K-deprived roots at 6 h by 5-fold and at 15 d by >6.5-fold. Additionally, *PbBCP1 (BLUE COPPER PROTEIN 1; Pbr010660.1*), a gene putatively coding for a protein associated with the cell wall, was up-regulated in K-deprived roots at 15 d after treatment by over 4-fold. Together, these data suggest the coordinated up-regulation of multiple candidates associated with the promotion of root growth and development under K deprivation. 

## 3. Discussion

Nutritional deficiencies alter the physiology and metabolism of plants over short-term and long-term periods of growth [37]. In response to nutrient deficiency, plants differentially regulate a plethora of genes and gene networks encoding a large number of proteins involved in acclimation and adaptation to the stress. Significant research has contributed to our understanding of the uptake, distribution, and homeostasis of potassium ions in model plants [38]. However, how xylophytes such as pear trees adapt to short-term and long-term nutrient deficiency is still not well understood. Previous work has demonstrated that plants respond rapidly to K deficiency [39]. However, as a long-term growing plant, pear trees can store significant quantities of K^+^, making it difficult to detect the status of K deficiency before symptoms are apparent. Upon the perception of K deficiency, the mobilization of K^+^ to reach the actively growing regions, changes in the root system architecture (RSA) through an increase in root elongation and angles, and changes in root K^+^-uptake mechanisms allow adaptation to the K deficiency and facilitate greater K^+^ acquisition [6]. Consistently, in this study with a pear rootstock, the redistribution of K^+^ within the shoots (Table 1) was observed along with increased root growth and coordinated changes in the root transcriptome to adapt to K deprivation.

**Figure 9:**
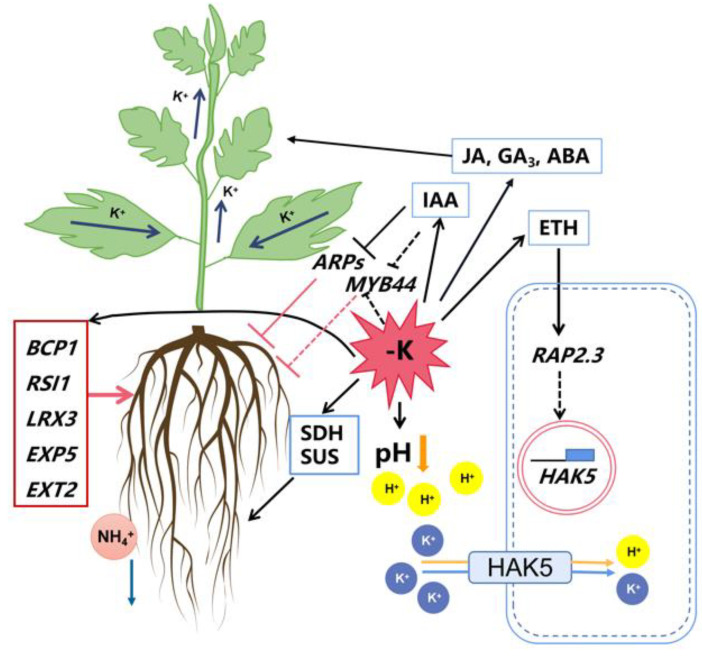
A proposed model to describe the effects of short-term K deprivation on root and shoot responses in *P. betulaefolia*. The model is based on physiological and root transcriptome responses described in this study. Dashed lines indicate putative effects. Solid lines indicate inferences made from the current study and from previous literature in other plant systems. Blue arrows indicate K^+^ transport and NH_4_^+^ efflux; red lines indicate effects on root growth.

In plants, K transporters and channels function in the absorption and translocation of K^+^ [23]. To date, many plant genes encoding K^+^ transporters and channels have been identified and found to mediate the uptake of K^+^ under low-K conditions [26,27]. A putative pear *HAK5*-related gene, *PbHAK5*, was up-regulated by up to 5-fold at 15 d after low-K stress. HAK5 is a member of the high-affinity K^+^ transporters in plants that likely functions as a K^+^–H^+^ symporter and is regulated by various factors such as K availability, ROS, and phytohormones [40]. The consistent and rapid reduction in rhizosphere pH in response to K deprivation (Figure 2B) suggests enhanced H^+^-ATPase activity to allow for the increased symport of K^+^, consistent with the up-regulation of *PbHAK5* expression. Together, these data suggest that a lack of K availability increases high-affinity transport systems in the low-K-tolerant pear rootstock (Figure 9). In other plants, different K^+^ channel family members are activated by changes in pH and contribute to the ABA-regulated closure of stomata, and the simultaneous promotion of root elongation under K deficiency [19,32]. Studies of *AKT1* and other K^+^ channel gene family members suggest the post-translational regulation of this group of channels [41]. Although changes in transcript abundance were not noted for the K^+^ channel family genes in the current study, they may yet play important roles in regulating pear responses to K stress, as the post-translational regulation of their activities may be operational. 

In the current study, the ethylene concentration was enhanced by over 2-fold in response to low-K stress, supporting a role for it in regulating K-deficiency stress responses. Ethylene is known to be involved in inducing the expression of *HAK5*, partly through ROS generation, and in inducing root-hair elongation in response to low-K stress [32,40]. Together, these may be expected to increase K^+^-uptake efficiency [27,32]. The production of ROS induced by low K in *Arabidopsis* is mediated by RCI3 (RARE COLD INDUCIBLE GENE3), a peroxidase that increases *AtHAK5* expression [27]. In our analysis, *PbRCI3 (Pb034488.2)* was only down-regulated at 6 h after treatment (Table S4). The ethylene-responsive AP2/ERF transcription factor *RAP2.11* is an important component of the low-K signaling pathway. AtRAP2.11 binds to the *AtHAK5* promoter, thereby enhancing its transcription [35]. Most of the ethylene-responsive transcription factors in the current study, including AP2/ERF family members, were rapidly down-regulated in response to low-K stress. However, a member of this family, *PbRAP2.3*, was up-regulated by K deprivation at 15 d after treatment. Similar to the function of RAP2.11, an increase in PbRAP2.3 abundance may enhance *PbHAK5* transcription. Alternatively, the increase in *PbRAP2.3* expression in response to an increase in ethylene may suggest a stress-related response that regulates root growth through increased aerenchyma formation, as noted in maize [18,42]. 

Commonly, transcription factors regulate the expression of stress-response genes and help plants in adapting to biotic and abiotic stress [43]. In Arabidopsis, MYB77 interacts with ARF7 to regulate lateral-root density under K deficiency, and its expression decreases under K deprivation [30]. In the current analyses, a putative MYB transcription factor, *PbMYB44*, displayed reduced transcript abundance (>7-fold) at 15 d after K deprivation in pear roots. It may be speculated that *PbMYB44* may similarly respond to enhanced IAA levels, allowing for plant acclimation to low-K stress by increasing root growth (Figure 7 and Figure 9 and Table S2). Interestingly, two *AUXIN REPRESSED PROTEINS* (*PbARP1* and *PbARP12.5*) displayed up to a 3.7-fold reduction in expression at 15 d after K deprivation, indicating a potential role for these genes in mediating the action of IAA in regulating root growth and K^+^ acquisition responses (Table S2). In tobacco, a loss of ARP1 function was associated with increased root and shoot growth [44]. Together, these data suggest that auxins may also play an important role in regulating root growth responses under K-deficiency stress in pears. 

In *Arabidopsis* and rice, lateral-root growth was reduced while root-hair elongation was enhanced in response to low-K stress [45]. In *Citrus*, an increase in root hairs was noted under K deficiency [46]. In tobacco, a low-K-tolerant genotype displayed reduced inhibitory effects on the root growth of first-order laterals under low-K stress [12]. In the current study, an increase in pear root growth was associated with the potentially increased translocation and metabolism of the key carbohydrate sources, sorbitol and sucrose, in the roots, as suggested by the increased transcript abundance of *SDH* and *SUS* genes [47,48]. An increase in root growth and potentially increased H^+^-ATPase activity to support high-affinity K^+^ acquisition require substantial inputs of additional energy. Furthermore, the NH_4_^+^ concentration in the medium was higher under low-K stress, even when the medium pH declined, suggesting enhanced NH_4_^+^ efflux (Figure 9) [49]. The enhanced cation efflux may add substantially to the energy demands of the roots [50]. Enhanced carbohydrate metabolism may likely support such energy demands for growth, nutrient homeostasis, and K^+^ acquisition in response to low-K stress. 

The transcriptome analysis also showed that many genes related to root elongation such as *PbBCP1*, *PbEXP5*, *PbEXT2*, *PbRSI1*, and *PbLEX3* are rapidly and strongly enhanced in expression in response to K deprivation. The increased abundance of their gene products may facilitate the enhanced root growth phenotype observed under K deprivation in pears. In the current study, few root hairs were observed in pear seedlings. We believe that this is likely due to differences in the mechanism of K-deficiency regulation in pear trees.

Potassium availability differentially impacts multiple metabolic and physiological processes, which coordinate plant adaptation to low-K conditions. The data from this study demonstrate that in the pear rootstock with a higher low-K tolerance, K deprivation leads to coordinated transcriptional changes that allow for sustaining root growth and for high-affinity K^+^ uptake (Figure 9). Additionally, K^+^ re-distribution within the shoot allows for continued new shoot growth. Together, these acclimation strategies enable the pear rootstock to survive and sustain its growth under low-K stress. Overall, this study establishes a solid foundation for understanding K-deficiency tolerance and provides potential candidate genes for future evaluation in pears.

## 4. Materials and Methods 

### 4.1. Plant Materials and Low-K Stress Treatment

Mature seeds of *P. betulaefolia* were surface sterilized and stratified in sand at 4 °C for approximately 50 d in a dark chamber. Seeds were considered as germinated when the cotyledons emerged from the sand. Germinated seedlings were transferred to pots containing vermiculite and placed in a greenhouse with 75% relative humidity and a 300 μmol m^−2^ s^−1^ light intensity for a 16/8 h light/dark photoperiod at 25 °C. Two treatments were initiated at the five-leaf stage using a hydroponic system; for the control treatment, full-strength Hoagland solution (containing 3 mM K^+^) was used, whereas in the second treatment, no K (solution containing 0 mM K) was used (Table S10). The treatments were set up in three replicates, with six plants in each replication. The seedlings were transferred into a 2 × 30 × 30 cm (with 1.5 L of medium) acrylic transparent container. This also allowed for the visualization of the root growth patterns under the two treatments. This experiment was sampled at two time-points—6 h and 15 d after the treatments were initiated—for the control and low-K treatments for root image analysis, morphology observation, and transcriptome sequencing.

### 4.2. Root Morphology of P. betulaefolia Seedlings 

The roots of control and treated *P. betulaefolia* seedlings were collected at 6 h and 15 d after the initiation of the experiment. The root system was photographed directly in an acrylic container and then processed in SmartRoot after PS (Photoshop CS6) processing. The root images were analyzed for total root length (RL), root surface area (RSA), average root diameter (RD), and root volume (RV) using the SmartRoot software (V5.0, Regent Instruments, QC, Canada) [51]. Data were collected from six replications per treatment.

### 4.3. ESI-HPLC-MS/MS Analysis of Phytohormones and ELISA Kit Analysis of Ethylene Content

The contents of IAA, GA_3_, ABA, JA, and SA were analyzed with high-performance liquid-chromatography electrospray tandem mass spectrometry (HPLC-ESI-MS/MS) [52]. The ETH content of the *P. betulaefolia* roots was detected using a plant hormone ETH ELISA kit (Meibiao Biology, China) [53].

### 4.4. Potassium and Nitrogen Concentrations of P. betulaefolia Seedlings

Seedlings were harvested at 15 d and separated into roots and shoots. K^+^ quantification was performed following the protocol described by Li (2018) [54]. The K content was determined by ICP-AES. The determination of total nitrogen (including NH_4_^+^-N and NO_3_^−^-N concentrations) in the plants was performed using a flow injection auto-analyzer (AA3, Seal Co., Bayern, Germany) [55].

### 4.5. RNA Extraction, Library Construction, and RNA-Seq

Roots of control and treated seedlings were collected at 6 h and 15 d after treatment, immediately frozen in liquid N_2_, and stored at −80 °C for further RNA-seq assays. Every RNA sample was derived from five independent seedlings. Total RNA was extracted from root samples using TRIzol reagent (Invitrogen, Carlsbad, CA, USA) [56]. To eliminate genomic DNA contamination, the RNA samples were treated with RNase-free DNase I (Takara, Tokyo, Japan). The quality of the RNA was checked using an Agilent 2100 RNA Bioanalyzer (Agilent, Santa Clara, CA, USA). Libraries for RNA-Seq were prepared by using the VAHTS mRNA-Seq v2 Library Prep Kit (Vazyme Biotech Co., Ltd., Nanjing, China). Sequencing was performed using a HiSeqXTen sequencing system using stranded paired-end 150 bp sequencing (Illumina, Inc., San Diego, CA, USA).

### 4.6. RNA-Seq Read Processing and Data Analysis

The original data obtained by high-throughput sequencing were converted into sequence data by CASAVA base calling. Each sample generated at least 6 gigabytes of data. For further analysis, the reads from the raw sequencing data were filtered, adaptors were removed, and low-quality (<Q30) reads trimmed. Then, the processed reads were mapped to the reference genome “Dangshansuli” (*P. bretschneideri Rehd.*) (http://peargenome.njau.edu.cn/default.asp?d=4&m=2) with Tophat2 with the following parameters: segment length, 25, and segment mismatches, 2. For the remaining parameters, the default settings were used [57]. The uniformity, insert length, and saturation of the sequencing data were analyzed based on the alignment results.

The quantitative analysis of gene expression was completed with Cufflinks based on RPKM (reads per kb per million reads). Differentially expressed genes (DEGs) were identified with an adjusted *p*-value < 0.05 for multiple tests, for controlling the false discovery rate [58]. Cluster analysis was performed on differentially expressed genes using a software cluster and Java Treeview. Gene functional annotations were based on the pear genome database and mapped onto GO terms. GO enrichment analysis was carried out using WEGO [59]. Kyoto Encyclopedia of Genes and Genomes (KEGG) pathways were identified according to *p*-values and adjusted *q*-values using a BLAST search against the KEGG database and were then mapped onto KEGG pathways [60].

### 4.7. Real-Time PCR Analysis

The RNA samples for the RNA-seq experiments (three biological replicates) were also used for real-time PCR assays to ensure the reliability and repeatability of the results. To eliminate genomic DNA contamination, total RNA was treated with DNase I (RNase Free) (Takara, Dalian, China). Then, the total RNA was used to synthesize cDNA in a reverse transcription reaction using random primers (10 μmol/L) (Promega, Madison, WI, USA). The cDNA samples were diluted to 4 ng/μL. Three biological replicates were analyzed using the Power SYBR Green PCR Master Mix (Applied Biosystems, Foster City, CA, USA) on a 7500 Real-Time PCR System machine (Applied Biosystems) according to the manufacturer’s protocol. The gene-specific primers were designed using the Primer 3 software [61]. The relative values for the expression levels were calculated by the 2^−ΔΔCt^ method [62]. The pear *Actin* (*Pbr024344.1*) gene was used as a control for expression analysis (Table S3).

## Figures and Tables

**Table 1:** Effects of low K on root morphology in *P. betulaefolia* seedlings at 15 d K deprivation.

Treatments	Length (cm)	Surf Area (cm^2^)	Diameter (mm)	Volume (cm^3^)	Tips
CK ^1^	417 ± 181.9 ^b^	239 ± 12.8 ^b^	10.1 ± 3.1 ^b^	12 ± 1.9 ^b^	71 ± 29.1 ^b^
LK	683 ± 79.6 ^a^	498 ± 166 ^a^	27 ± 3.7 ^a^	35 ± 18.6 ^a^	164 ± 48.1 ^a^

^1^ CK: control; LK: Low-K treatment. Similar letters after numbers indicate that values are not significantly different within the column.

## Supplementary Materials

The supplementary materials can be found at https://www.mdpi.com/1422-0067/21/22/8857/s1.
