# Supplementary material for: Transcriptome Analysis of Pyrus betulaefolia Seedling Root Responses to Short-Term Potassium Deficiency
Source: Int J Mol Sci. 2020 Nov 23;21(22):8857. doi: 10.3390/ijms21228857 (PMC7700257; doi:10.3390/ijms21228857)
Supplement: Supplementary file 1 [file ijms-21-08857-s001.zip › appendices/Table 1.doc]

**Table 1 Effects of low K on root morphology of *P. betulaefolia* seedlings**

| **Treatments** | **Length (cm)** | **Surf area (cm^2^)** | **Root diam (mm)** | **Root volume (cm^3^)** | **Tips** |
| --- | --- | --- | --- | --- | --- |
| **CK** | **417±181.9^b^** | **239±12.8^b^** | **10.1±3.1^b^** | **12±1.9^b^** | **71±29.1^b^** |
| **LK** | **683±79.6^a^** | **498±166^a^** | **27±3.7^a^** | **35±18.6^a^** | **164±48.1^a^** |
