# Supplementary material for: Transcriptome Analysis of Pyrus betulaefolia Seedling Root Responses to Short-Term Potassium Deficiency
Source: Int J Mol Sci. 2020 Nov 23;21(22):8857. doi: 10.3390/ijms21228857 (PMC7700257; doi:10.3390/ijms21228857)
Supplement: Supplementary file 1 [file ijms-21-08857-s001.zip › appendices/Table. S1.doc]

**Table S1 Summary of RNA-seq quality information**

| Sample name | Raw reads | Clean reads | Clean bases | Error rate (%) | Q20 (%) | Q30 (%) | GC content (%) | Total mapped  （%） | Exon (%) | Intron (%) | Intergenic (%) |
| --- | --- | --- | --- | --- | --- | --- | --- | --- | --- | --- | --- |
| CK _1 | 56905884 | 55376420 | 8.31G | 0.03 | 96.69 | 91.05 | 47.46 | 70.11 | 97.2 | 1.1 | 7.7 |
| CK _2 | 46290190 | 45329512 | 6.8G | 0.03 | 96.61 | 90.86 | 47.69 | 67.95 | 90.3 | 1.4 | 8.3 |
| CK _3 | 53464324 | 51822240 | 7.77G | 0.03 | 96.66 | 90.98 | 47.52 | 67.12 | 90.5 | 1.4 | 8.1 |
| LK6 h _1 | 50686310 | 49586148 | 7.44G | 0.03 | 96.54 | 90.72 | 47.19 | 70.40 | 89.8 | 1.7 | 8.5 |
| LK6 h _2 | 52750306 | 51448264 | 7.72G | 0.03 | 96.85 | 91.35 | 47.36 | 70.13 | 90.2 | 1.4 | 8.3 |
| LK6 h _3 | 50656266 | 49348666 | 7.4G | 0.03 | 96.47 | 90.63 | 47.63 | 71.60 | 90.7 | 1.4 | 7.9 |
| LK15 d _1 | 41761754 | 41070562 | 6.16G | 0.03 | 97.78 | 93.97 | 47.05 | 75.26 | 90.2 | 1.8 | 8.0 |
| LK15 d _2 | 52086374 | 50913662 | 7.64G | 0.03 | 97.63 | 93.67 | 47.02 | 75.36 | 89.9 | 1.8 | 8.3 |
| LK15 d _3 | 56162998 | 55045168 | 8.26G | 0.03 | 97.71 | 93.86 | 46.88 | 74.89 | 89.8 | 1.9 | 8.3 |
