# Supplementary material for: Transcriptome Analysis of Pyrus betulaefolia Seedling Root Responses to Short-Term Potassium Deficiency
Source: Int J Mol Sci. 2020 Nov 23;21(22):8857. doi: 10.3390/ijms21228857 (PMC7700257; doi:10.3390/ijms21228857)
Supplement: Supplementary file 1 [file ijms-21-08857-s001.zip › appendices/Table. S10.doc]

**Table S10** The composition of nutrient solution formula

| **Content** | **CK (mg/L)** | **Content** | **LK (mg/L)** |
| --- | --- | --- | --- |
| Ca(NO_3_)_2_·4H_2_0 | 945 | Ca(NO_3_)_2_·4H_2_0 | 945 |
| KNO3 | 506 | NH_4_NO_3_ | 240 |
| KH_4_NO_3_ | 80 | (NH_4_)H_2_PO_4_ | 115 |
| KH_2_PO_4_ | 136 | MgSO_4_ | 241 |
| MgSO_4_ | 241 | - | - |
| FeNaEDTA | 36.7 | FeNaEDTA | 36.7 |
| KI | 0.83 | NaI | 0.75 |
| H_3_BO_3_ | 6.2 | H_3_BO_3_ | 6.2 |
| MnSO_4_·H_2_0 | 16.9 | MnSO_4_·H_2_0 | 16.9 |
| ZnSO_4_·7H_2_0 | 8.6 | ZnSO_4_·7H_2_0 | 8.6 |
| Na_2_MoO_4_·2H_2_0 | 0.25 | Na_2_MoO_4_·2H_2_0 | 0.25 |
| CuSO_4_·5H_2_0 | 0.025 | CuSO_4_·5H_2_0 | 0.025 |
| CoCl_2_·6H_2_0 | 0.025 | CoCl_2_·6H_2_0 | 0.025 |
| pH(25℃) | 5.8 | pH(25℃) | 5.8 |
