# Supplementary material for: Transcriptome Analysis of Pyrus betulaefolia Seedling Root Responses to Short-Term Potassium Deficiency
Source: Int J Mol Sci. 2020 Nov 23;21(22):8857. doi: 10.3390/ijms21228857 (PMC7700257; doi:10.3390/ijms21228857)
Supplement: Supplementary file 1 [file ijms-21-08857-s001.zip › appendices/Table. S2.doc]

**Table S2** **List of important DEGs in response to low-K stress**

| Gene | Gene ID | Log2 (LK/CK)^a^ | | 注释 |
| --- | --- | --- | --- | --- |
|  |  | LK-6h vs CK | LK-15d vs CK | Annotation |
| *Pbr037826.1* | LOC103950380 | 0.66736 | 2.32888 | PbHAK5 |
| *Pbr037395.1* | LOC103950289 | 1.17246 | 2.01637 | PbSUS |
| *Pbr032776.1* | LOC103933318 | 1.35665 | 3.05121 | PbSDH1.1 |
| *Pbr032775.1* | LOC103933317 | 1.10663 | 1.68231 | PbSDH1.2 |
| *Pbr013913.1* | LOC103960508 | 1.03573 | 1.99795 | PbSDH1.3 |
| *Pbr032777.1* | LOC103933319 | 0.67157 | 1.32940 | PbSDH1.4 |
| *Pbr024532.1* | LOC103927256 | 2.33672 | 2.71132 | PbRSI1 |
| *Pbr016594.1* | LOC103962730 | 2.71896 | 3.23473 | PbLRX3 |
| *Pbr039073.1* | XM_009362050.2 | 2.03250 | 2.68214 | PbEXP5 |
| *Pbr031110.1* | LOC103932085 | 0.95546 | 2.63264 | PbEXT2 |
| *Pbr010660.1* | LOC103958339 | 0.80921 | 2.02205 | PbBCP |
| *Pbr019150.1* | LOC103944840 | 1.84289 | 2.63398 | PbTIP2.1 |
| *Pbr009158.1* | LOC103957395 | -0.57665 | -2.07553 | PbPIP1.1 |
| *Pbr042254.1* | LOC103939039 | 1.41587 | 3.16134 | PbPruav1 |
| *Pbr000063.1* | LOC103944341 | 2.02389 | 2.48044 | PbHevb5 |
| *Pbr011136.1* | LOC103959040 | 1.85189 | 1.46128 | PbPSPC13 |
| *Pbr031118.1* | LOC103932068 | 3.12189 | 3.94449 | PbDIR1 |
| *Pbr035599.1* | LOC103949587 | 2.05878 | 3.58263 | PbDIR1.2 |
| *Pbr004997.1* | LOC103954933 | -0.57706 | -3.18563 | PbnsLtp8 |
| *Pbr003247.1* | LOC103940416 | 0.88129 | 1.75465 | PbSEN21 |
| *Pbr015413.1* | LOC103961717 | 1.24838 | 1.72834 | PbNDR1/H1N1 |
| *Pbr030796.1* | LOC103931888 | 1.22846 | 1.22376 | PbMLP28 |
| *Pbr034452.1* | LOC103934314 | -0.63190 | -1.46362 | PbEF1 |
| *Pbr041719.1* | LOC103952130 | -1.16393 | -1.81313 | PbEF1-2 |
| *Pbr026725.1* | LOC103928880 | -0.72338 | -1.32058 | PbV-ATPaseE |
| *Pbr012197.2* | HM044853.1 | -0.77999 | -2.65720 | PbDFN1 |
| *Pbr011839.1* | LOC103959223 | -0.81262 | -1.5277 | PbRIGIDA4 |
| *Pbr014868.1* | LOC103943662 | -0.93903 | -1.53652 | PbTCTP |
| *Pbr009462.1* | LOC103936226 | -1.16518 | -2.26366 | PbHSP18.5 |
| *Pbr034275.2* | LOC103949106 | -1.49371 | -4.18969 | PbTIL1 |
| *Pbr040482.1* | LOC103937807 | -1.67672 | -3.52909 | PbCs120 |
| *Pbr040484.1* | LOC103937812 | -2.40361 | -4.41741 | PbDHN3 |
| *Pbr023558.1* | LOC103926590 | 1.86734 | 2.85364 | PbPRPDC2.15 |
| *Pbr023676.2* | LOC103926555 | 1.38153 | 2.52093 | PbPRPDC2.15-like |
| *Pbr041603.1* | LOC103938607 | 1.67638 | 2.42639 | PbPRPDC2.15-like |
| *Pbr041827.1* | LOC103938713 | 0.85821 | 2.8613 | PbPX16 |
| *Pbr008178.1* | LOC103956697 | 2.36127 | 3.27342 | PbNADH |
| *Pbr016849.1* | LOC103944343 | 0.76347 | 2.40664 | PbCBR1 |
| *Pbr042101.1* | LOC103952374 | 0.93520 | 1.96765 | PbFDX-NADH |
| *Pbr040385.1* | LOC103937987 | -0.23371 | -1.126 | PbFDX-NR |
| *Pbr001154.1* | LOC103939427 | 0.76796 | 1.48088 | PbFDX-RB1 |
| *Pbr037590.1* | LOC103936374 | 1.18933 | 1.43058 | PbAP1 |
| *Pbr001543.2* | LOC103952863 | 1.44102 | 2.47901 | PbLDOX |
| *Pbr013510.1* | LOC103943352 | 1.08190 | 0.72602 | PbCOMT3 |
| *Pbr019531.1* | LOC103965055 | 1.44064 | 2.37468 | PbCHS |
| *Pbr003752.1* | LOC103940646 | 1.32600 | 2.28134 | PbCHI3 |
| *Pbr014749.1* | LOC103943555 | 1.28593 | 2.73337 | PbGS |
| *Pbr019533.1* | LOC103965057 | 1.13855 | 1.84699 | PbPKS5 |
| *Pbr016887.1* | LOC103944383 | 0.99244 | 1.09427 | PbMTR |
| *Pbr026727.1* | LOC103928882 | 0.83386 | 1.57298 | Pb6PGDH |
| *Pbr005931.1* | LOC103928717 | 0.79092 | 1.67979 | PbDFR/FNR |
| *Pbr022368.1* | LOC103966943 | 0.77534 | 1.98714 | PbADC |
| *Pbr016485.1* | LOC103962559 | 0.66678 | 0.66897 | PbAdoHcyase |
| *Pbr018303.1* | LOC103964280 | 0.88493 | 1.81606 | PbCYP71A1 |
| *Pbr015176.1* | LOC103961467 | -0.82842 | -1.65698 | PbCYP78A5 |
| *Pbr012891.1* | LOC103960076 | -0.94218 | -2.06329 | PbH2A1 |
| *Pbr029415.2* | LOC103930957 | -1.04542 | -2.49463 | PbH1 |
| *Pbr004201.3* | LOC103967337 | -0.88982 | -2.04940 | PbSUI1-2-like |
| *Pbr033681.1* | LOC103948771 | -1.05292 | -1.82049 | PbSUI1-2 |
| *Pbr029941.1* | LOC103947231 | -0.58394 | -1.29408 | PbFUS |
| *Pbr015431.1* | LOC103961819 | -0.91599 | -1.57491 | PbRBP45C |
| *Pbr033635.1* | LOC103948736 | -0.83997 | -1.51504 | PbRBP47 |
| *Pbr028963.1* | LOC103930608 | -1.04047 | -3.40623 | PbDAS3 |
| *Pbr033373.1* | LOC103933608 | -1.05102 | -1.94455 | PbDnaJ |
| *Pbr006874.1* | LOC103955508 | -1.13465 | -3.81647 | PbPRP36.4 |
| *Pbr009528.1* | LOC103936790 | -1.23493 | -3.42726 | PbPI-PLC2 |
| *Pbr015309.1* | LOC103961604 | -0.99135 | -2.92609 | PbMYB44 |
| *Pbr012024.1* | LOC103959375 | 0.91703 | 1.11670 | PbRAP2.3 |
| *Pbr000392.1* | KC422235.1 | -0.52718 | -2.52904 | PbARP1 |
| *Pbr016230.1* | LOC103944186 | -0.88937 | -3.72009 | PbARP12.5 |
| *Pbr014779.1* | LOC103943587 | 0.95301 | 2.55166 | Pbr014779.1 |
| *Pbr030179.1* | LOC103931441 | -1.22924 | -1.64460 | Pbr030179.1 |
| *Pbr020285.1* | LOC103938811 | -1.65022 | -3.62164 | Pbr020285.1 |
| *Pbr030334.1* | LOC103947691 | 1.79738 | 2.86933 | PbNRT2.4 |
| *Pbr030335.1* | LOC103947692 | 1.70507 | 2.85815 | PbNRT2.4-like |

Note: ^a^The fold change of each gene was transformed into “log2”. The genes with log2(LK/CK)>1 were defined as signifianty up-regulated; The genes with log2(LK/CK)<-1 were defined as significantly down-regulated.
