# Supplementary material for: Transcriptome Analysis of Pyrus betulaefolia Seedling Root Responses to Short-Term Potassium Deficiency
Source: Int J Mol Sci. 2020 Nov 23;21(22):8857. doi: 10.3390/ijms21228857 (PMC7700257; doi:10.3390/ijms21228857)
Supplement: Supplementary file 1 [file ijms-21-08857-s001.zip › appendices/Table. S3.doc]

**Table S3 Primer sequences used in qRT-PCR**

| Genes | Forward primer (5’-3’) | Reverse primer (5’-3’) |
| --- | --- | --- |
| *PbRSI1* | CGAGATGTAAGAACCGTTG | CCAGTTGTTGTAGCAAGG |
| *PbEXP5* | CGAGATTAGGTGTGTGAACG | AAGACAGGCTGAGAGAGGTC |
| *PbBCP1* | TCCACAGCAGCACAGAAA | ACAAGAAGGTCACCAACGG |
| *PbSDH1* | TGGCACCGTCAAAGTTTC | CATTCCGACAAGGCAGACT |
| *PbHAK5* | GAACCAGCAACTGAAGGA | TACACATAGAGCGGAGACG |
| *PbMYB44* | CAGCAGTAACAGCGAGAA | TCCTCACCTCCTTCCTTA |
| *Actin* | TGGGCTTTGCTCCTCTTAC | CCTTCGTGCTCATCTTACC |
